# Umbilical cord mesenchymal stromal cells—from bench to bedside

**DOI:** 10.3389/fcell.2022.1006295

**Published:** 2022-10-12

**Authors:** Shashank Chetty, Reza Yarani, Ganesh Swaminathan, Rosita Primavera, Shobha Regmi, Sravanthi Rai, Jim Zhong, Abantika Ganguly, Avnesh S Thakor

**Affiliations:** ^1^ Interventional Radiology Innovation at Stanford (IRIS), Stanford University, Department of Radiology, Palo Alto, CA, United States; ^2^ Translational Type 1 Diabetes Research, Department of Clinical, Research, Steno Diabetes Center Copenhagen, Gentofte, Denmark; ^3^ Department of Diagnostic and Interventional Radiology, Leeds Teaching Hospitals NHS Trust, Leeds, United Kingdom

**Keywords:** umbilical cord (UC), mesenchymal stem cells, extracellular vesicles (EVs), clinical trials, regenerative therapy, single cell transcriptomics

## Abstract

In recent years, mesenchymal stromal cells (MSCs) have generated a lot of attention due to their paracrine and immuno-modulatory properties. mesenchymal stromal cells derived from the umbilical cord (UC) are becoming increasingly recognized as having increased therapeutic potential when compared to mesenchymal stromal cells from other sources. The purpose of this review is to provide an overview of the various compartments of umbilical cord tissue from which mesenchymal stromal cells can be isolated, the differences and similarities with respect to their regenerative and immuno-modulatory properties, as well as the single cell transcriptomic profiles of *in vitro* expanded and freshly isolated umbilical cord-mesenchymal stromal cells. In addition, we discuss the therapeutic potential and biodistribution of umbilical cord-mesenchymal stromal cells following systemic administration while providing an overview of pre-clinical and clinical trials involving umbilical cord-mesenchymal stromal cells and their associated secretome and extracellular vesicles (EVs). The clinical applications of umbilical cord-mesenchymal stromal cells are also discussed, especially in relation to obstacles and potential solutions for their effective translation from bench to bedside.

## Introduction

Mesenchymal stromal cell (MSC) therapy has enormous potential in regenerative medicine given their paracrine, anti-inflammatory and immunomodulatory capabilities. Although, several studies in early 2000s reported the ability of UC-MSCs to differentiate into various cell types, these claims were primarily based on *in vitro* experiments with limited characterization (Caplan, 1991; Guadix et al., 2017; Viswanathan et al., 2019). In 2006, the International Society for Cellular Therapy (ISCT) established the minimal criteria required for a cell to be classified as an MSC: 1) They must adhere to the surface in a tissue culture plate, 2) express adhesion markers CD44 (hyaluronan receptor), mesenchymal markers such as CD90 (thy-1) and CD105 (endoglin), and lack expression of hematopoietic markers e.g. CD34, 3) differentiate into multiple lineages *in vitro*, 4) self-renew and exhibit low immunogenicity (Dominici et al., 2006). Over several years, MSCs have gained much attention as a potential therapeutic for several clinical applications such as osteoarthritis, myocardial infarction, graft-versus-host disease (GVHD), diabetes mellitus, multiple sclerosis, systemic lupus erythematosus, kidney injury, amyotrophic lateral sclerosis, spinal cord injury and several other diseases (Parekkadan and Milwid, 2010; Saeedi et al., 2019; Payandeh et al., 2022; Norooznezhad et al., 2022; Ullah et al., 2019). In addition, MSCs release soluble factors, extracellular vesicles (EVs) such as exosomes, and microvesicles (MVs) that can collectively act in a paracrine and autocrine fashion to repair tissue and modulate the tissue microenvironment (Joerger-Messerli et al., 2018). Stromal cells derived from the embryos, such as those resulting from the inner cell mass, are surrounded by ethical issues related with embryo destruction. While induced pluripotent stem cells circumvent the ethical issues, they introduce risk of tumor formation by transferring pluripotent genes (Lo and Parham, 2009). In contrast, MSCs that can be isolated from adults (bone marrow, peripheral blood, adipose tissue and dental pulp) and birth-related tissues (cord blood, Amnion membrane and amniotic fluid) have low to non-existent tumorigenic potential which, combined with absence of the major histocompatibility complex II and low expression of co-stimulatory factors CD80, CD86 and CD40, allow for allogeneic administration of MSCs across a wide range of clinical applications (Chen et al., 2004; Lazarus et al., 2005; Fouillard et al., 2007; Lee et al., 2008). Therefore, the umbilical cord (UC), which is considered medical waste and retrieved at delivery using a painless, simple and safe approach, has piqued significant interest in recent years as a potential source for MSCs. The existing isolation procedure for MSCs has consistently resulted in effective collection of substantial quantities of MSCs from UC tissues. Furthermore, UC-MSCs also have a greater self-renewal capacity (300 cell divisions) and a shorter doubling time (30–36 h) (Koike et al., 2014). Clinical trials have shown that UC-MSCs can heal nerve, bone, heart and kidney injuries, as well as promote the formation of blood vessels; these finding have been reported in several clinical trials as reported in the ClinicalTrials.gov database–please see Table 3 for full details (Lv et al., 2013; Chang et al., 2014; Kim et al., 2015; Bartolucci et al., 2017; Riordan et al., 2018; Powell and Silvestri, 2019; He et al., 2020; Özmert and Arslan, 2020; Zhang et al., 2021; Kim et al., 2021; Lee et al., 2021; Shi et al., 2021; Monsel et al., 2022; Zang et al., 2022). The main benefit of UC-MSCs over MSCs from other sources is their availability as well as their high proliferation and plasticity (Pipino et al., 2013).

The purpose of this review is to provide an overview of different UC tissue compartments to isolate MSCs, the characteristic differences and similarities in their regeneration and immuno-modulatory factors, as well as the single cell transcriptomic profiles of *in vitro* grown and tissue extracted UC-MSCs. The review also highlights the therapeutic potential and biodistribution of UC-MSCs after systemic administration in addition to an overview of completed pre-clinical trials using UC-MSCs, and therapeutic interventions using the secretome and extracellular vesicles (EVs). Finally, the banking and socio-ethical issues concerning UC-MSCs for future healthcare needs are discussed.

## Umbilical cord mesenchymal stromal cells

**FIGURE 1:**
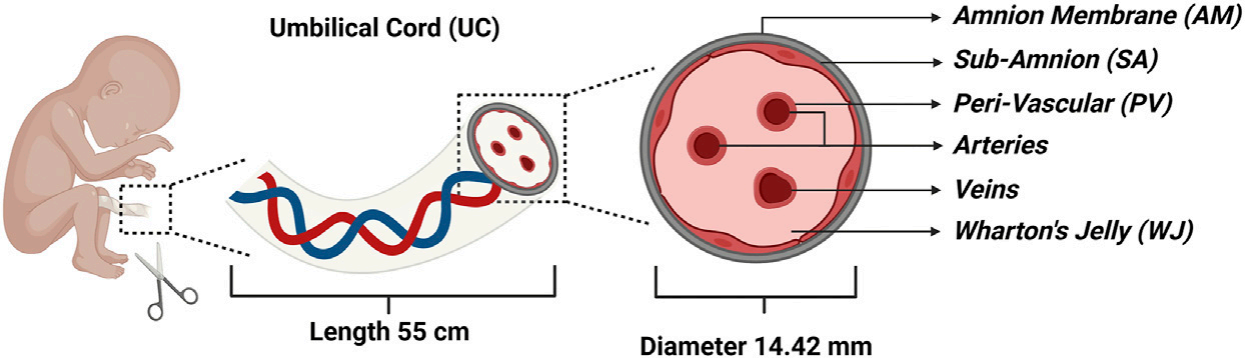
Schematic illustration of the umbilical cord (UC) representing the four major compartments: Amnion Membrane (AM), Wharton’s Jelly (WJ), Sub-Amnion (SA), and Peri-Vascular (PV) region along with arteries and veins.

MSCs isolated from various areas of the UC provide a possible alternative for BM and adipose tissue (AD) derived MSCs, as both involve invasive isolation procedures, and have a low cell recovery and shorter stemness (Baksh et al., 2007). UC is a conduit that connects the mother and baby in the pregnancy and is made of narrow-tube structures that prevents the cord itself from compressing, bending, or twisting. The UC develops between the 4th and 8th week of human embryogenesis, with a mean length of 55 cm, mean diameter of 14.42 mm, and mean weight of 40 g (32). UC consists of two arteries and one vein encased in the proteoglycan-rich gelatinous Wharton’s jelly (WJ) and associated by the amnion layers. A schematic illustration of the UC is shown in Figure 1. The extracellular matrix is rich in fibroblast growth factors 1 and 2, platelet-derived growth factor (PDGF), insulin-like growth factor 1 (IGF-1), epidermal growth factor (EGF) and transforming growth factor (TGF). In contrast to other MSC tissue sources, the colony forming unit-fibroblasts (CFU-F) frequency during collection is higher from the UC [(Davies et al., 2017), (Majore et al., 2009)].

Commonly, MSC isolation from UC is divided into four main sections: the Wharton’s Jelly (WJ), the perivascular area (PV), the sub-amnion (SA), and the Amnion membrane (AM). WJ-MSCs are isolated by dissecting out the arteries and extracting the WJ from the UC using knife or forceps. PV-MSCs are separated by looping the collected blood arteries and enzymatically digesting them to allow cell dissociation for further culture. SA-MSCs are isolated by dissecting the cord lining using scissors or a scalpel and then cultivating the explants to get adherent cells. AM-MSCs are isolated by dissecting the UC and dipping the Amnion membrane side in an enzymatic solution for cell dissociation and subsequent expansion in culture medium (Subramanian et al., 2015). McElreavey *et al.* isolated fibroblast-like cells that showed features of typical MSCs from the WJ of UC for the first time in 1991 (McElreavey et al., 1991). In 2003, Mitchell *et al.* extracted MSCs from WJ through explant culture and differentiated them into glial cells and neurons as a possible alternative for regenerative treatments (Mitchell et al., 2003). The first cord blood transplantation was conducted in France in 1988 using MSCs, as they were suggested to be circulating in the pre-term fetus (Gluckman et al., 1989). Romanov *et al.* developed an effective isolation prototype of MSCs from UC sub-endothelial layers, after which UC became increasingly prevalent as a potential source of MSCs (Romanov et al., 2003).

## Characterization of umbilical cord-mesenchymal stromal cells

Cross-sectional histological staining of UC with hematoxylin and eosin reveals six different regions: arteries, veins, PV, WJ, AM, and SA. Among them, WJ takes up a significant amount of the UC and includes a greater number of MSCs with stellate-like shape than the other regions; up to 5.5 × 10^6^ (Subramanian et al., 2015). MSC isolation from PV, SA and AM, requires enzymatic treatment and repeated culture because these areas are closely linked through the extracellular matrix due to which cross contamination of MSCs from these compartments is possible (Zhang et al., 2012). In contrast, isolation of MSCs from WJ is simpler and faster because of their short doubling time, allowing sufficient cell counts to be achieved in a shorter culture period for clinical translation, thereby reducing the risk of culture-induced genetic manipulation and microbial contamination (Liau et al., 2020). The WJ-MSCs have higher viability and proliferation rates than other areas (i.e. AM, SA, PV). Although the telomerase activity of MSCs from WJ, AM, SA, and PV are comparable, the regenerative ability of WJ-MSCs is substantially higher than those from the other areas at higher passages (Pera, 2009). MSCs generated from WJ, AM, SA, and PV, have been shown to easily develop into adipocytes, chondrocytes, and osteocytes (Subramanian et al., 2015). However, the osteogenic differentiation of WJ-MSCs presents greater Von Kossa-stained cells, revealing increased calcium accumulation, whereas the chondrogenic differentiation of WJ- and PV-MSCs shows Alcian blue-stained cells, revealing higher proteoglycan and glycosaminoglycan synthesis by articular chondrocytes (Mitchell et al., 2003). Additionally, gene expression of osteogenic markers (OCN, OPN, ALP, BSP) in WJ-MSCs is considerably greater (5-fold) than other areas (AM, SA, PV). Similarly, chondrogenic marker gene expression (COL2A1, COMP, FMOD, SOX9) is substantially greater in WJ-MSCs (30-fold) (Subramanian et al., 2015).

Immuno-phenotyping of MSCs isolated from various compartments of UC revealed positive for CD29, CD90, CD44, CD73, HLA-ABC, CD105 and negative for CD34, CD14, CD45, CD19, HLA-DR ABC, CD117. WJ-MSCs have significant levels of CD29, HLA-ABC, CD44, and CD73 markers when compared to cells from AM, SA, and PV (Subramanian et al., 2015). WJ-MSCs also have higher levels of the CD24 and CD108 markers that distinguish MSCs from non-MSCs (Wetzig et al., 2013). The pericyte markers CD146 and CD271 are positive in both WJ and PV-MSCs, indicating that there is no apparent anatomical divide between the two areas (Covas et al., 2008). In contrast, CD140b and CD49 days are markers that are infrequently expressed in AM and SA-MSCs owing to contamination with neighboring fibroblast or stromal cells during isolation (Mafi et al., 2011). The expression of the CD40 marker, which is found in non-MSCs, is reported in MSC cultures primarily isolated from AM, SA, PV (50.8%–70.2%) and least from WJ (26.1%–27.4%); likely due to cross-contamination of smooth muscle, epithelial, endothelial and fibroblast cells (Majore et al., 2011). The immuno-histological labelling and RT-PCR studies of MSCs generated from various compartments of UC are positive for pluripotent markers Tra-1–60, Tra-1–81, SSEA-4, SSEA-3, as well as pluripotent genes OCT4A, SOX2, OCT2, OCT4B, OCT1 and NANOG. Pluripotent genes are highly expressed in WJ-MSCs, while fibroblast-associated genes FAP and FSP, are significantly expressed in AM- and SA-MSCs (Subramanian et al., 2015). Despite high levels of expression of pluripotent markers, researchers have revealed that UC-MSCs do not form teratomas when injected into immunodeficient SCID mice (Wang et al., 2013; Chen et al., 2014).

It has been observed that MSCs isolated from various compartments of UC have distinct morphologies. WJ-MSCs form a confluent monolayer of adherent cells with stellate-like morphology, while MSCs obtained from AM, SA, and PV areas show fibroblast-like morphology *in vitro* (Subramanian et al., 2015). MSCs generated from the entire UC showed colonies with both epithelioid and fibroblast morphologies. Further differences in the MSCs properties isolated from the different UC compartments are compared in Table 1 (Zhang et al., 2012; Subramanian et al., 2015).

**TABLE 1:** Comparison of MSC characteristics isolated from the different sections of umbilical cord.

Property/Feature	Wharton’s jelly	Perivascular	Sub-amnion	Amnion
Morphology	Stellate-like	Fibroblast-like		
No. of cells in primary cell culture	4.9–6.6 × 10^6^	2.8–3.6 × 10^6^	2.3–3.0 × 10^6^	1.8–2.2 × 10^6^
Cell proliferation— Optical density (passage)	3.5 (P3), 3.9 (P5), 3.8 (P10)	2.8 (P3), 3.1 (P5), 2,9 (P10)	2.1 (P3), 2.4 (P5), 2,4 (P10)	1.9 (P3), 2.5 (P5), 2.4 (P10)
Telomerase activity— (passage)	20 (P1), 18 (P10)	19 (P1), 16 (P10)	18 (P1), 13 (P10)	18 (P1), 14 (P10)
Cell viability—Optical density (passage)	1.3 (P3), 1.4 (P5), 1.3 (P10)	1.0 (P3), 1.1 (P5), 1.1 (P10)	0.7 (P3), 0.9 (P5), 0.8 (P10)	0.6 (P3), 0.8 (P5), 0.9 (P10)
Differentiation potential	Adipocyte differentiation with enhancing osteo- and chondrogenesis	Osteo- and adipocyte differentiation with enhancing chondrogenesis	Exhibit adipo-, osteo- and chondrogenic differentiation	
Pericyte markers	Express CD146 and CD271		Do not express CD146 and CD271	
Cross-cell contamination (fibroblast, stromal cells)	Do not express CD140b and CD49 days surface markers		Express CD140b and CD49 days surface markers	
Fibroblast-specific marker	Do not express FAP and FSP gene		Express FAP and FSP gene	
Surface marker expression	Enhance expression of CD29, HLA-ABC, CD44 and CD73 surface markers	Positive for CD29, CD90, CD44, CD73, HLA-ABC, CD105 and negative for CD34, CD14, CD45, CD19, HLA-DR ABC, CD117 surface markers		
MSCs (CD24 and CD108) vs*.* Non-MSCs (CD40)	Express CD24 and CD108	Express CD40 marker from smooth muscle, epithelial, endothelial, or fibroblast cells		
Pluripotency expression	Enhance expression of pluripotency markers and gene	Positive for pluripotent markers *i.e.* Tra-1–60, Tra-1–81, SSEA-4, SSEA-3 and pluripotent gene i.e. OCT4A, SOX2, OCT2, OCT4B, OCT1, NANOG		

The hypothesis that all UC-MSCs have the same therapeutic qualities and functions ignores biological heterogeneity and may limit their use in disease treatment (Wegmeyer et al., 2013). The standardization of UC-MSC populations is one of the most important criteria influencing their clinical efficacy. Using routine analysis of cell surface antigen expression and differentiation potential, a better understanding of the differences in functional properties of UC-MSC from different donors, tissues, clones and even different cells from the same clonal population can be achieved. However, global transcriptional profiling can clearly reveal expression patterns that identify biological states or compare genes with comparable expression patterns (Wells and Choi, 2019). Bulk transcriptomic profiles, however, lack the spatial and temporal precision to differentiate genetic variability among low colonized groups within the same tissue of origin, unlike single cell transcriptomics which can resolve subtle changes across *in vitro* expanded clonal UC-MSC populations (Wang et al., 2021).

Regarding molecular heterogeneity within *in vitro* expanded UC-MSCs, Huang *et al.* revealed distinct subpopulations of cultured UC-MSCs from the same tissue source, defined by differential expression of genes (Huang et al., 2019). Interestingly, the expression pattern of the sub-populations remained persistent throughout passage numbers, donors, and following both INFγ and TNFα stimulation. Similar conclusions have been drawn by other researchers regarding variability of sub-populations among different MSC sources (AD, BM and UC) (Hou et al., 2021) and genomic heterogeneity across subpopulations of cultured UC-MSCs using gene markers for each sub-population as shown in Table 2 (Hou et al., 2021) (Barrett et al., 2019).

**TABLE 2:** Genomic heterogeneity among the subpopulation of cultured UC-MSCs.

Type	Genomic markers
Common Clusters	*BRCA1, CDCA5, HMGB1, HMMR, MELK, PRC1, RACGAP1, AREG, CSF3, CCL20, IL6*
Subpopulations in UC-MSC	
P1	*MKI67, TPX2, TOP2A, HMGA1, HMGA2, HMGB2, TFDP1, MYBL1*
P2	*CNN3, PTTG1, HMGB1, TUBA1A, TUBB4B, TUBA1C*
P3	*IGFBP5, IGFBP3, COL3A1, TGFBI, SOX4, FOS, MMP2, CCl2, GJA1, SOD2*
Tissue and *in vitro* expanded UC-MSC	
Tissue	*BMP2, RGCC, BAMB1, SNAL1, CTNNB1, TGFBR2, COL1A1, TGFB2, PLAU, ITGAU*
*In vitro* expanded	*CCT3, CCT6A, CCT2, RUVBL2, RUVBL1, NHP2, CCT8, CCT7, NOP10, CCT5, POLD2, NDK, APEX1*
Tissue-extracted UC-MSC	
P1- stromal	*COL1A2, COL6A1, COL4A1, CD83, CXCL2, HSPA1B, IL24, CCL16, MK167, RRM2, CTSK*
P2- pericyte	*RGS5, CD36, MCAM*
P3- epithelial	*CDH1, KRT7, KRT17, PERP, SFN, KRT14, KRT13*

Several studies noted there was a correlation between the weighted percentage of these subpopulations in the clonal line and their immunomodulatory effects (Barrett et al., 2019; Huang et al., 2019; Hou et al., 2021; Wang et al., 2021). For example, HMGB2 and other genes are highly expressed in UMSC1 subpopulations that is believed to contribute to their proliferative capacity, while genes that are highly expressed in UMSC3 subpopulations align towards a committed differentiation status (Wang et al., 2021). On the other hand, high expression of AREG, CSF3, CCL20 and IL6 make UC-MSCs exert a stronger immunomodulatory effect compared to other clonal lines or even other sources of MSC (Hou et al., 2021). In addition, it was also observed that the limited heterogeneity observed among UC-MSCs was related to cell cycle stages, with the CD168 marker being negatively corelated with the immunosuppressive effect of those subpopulations of MSCs (Huang et al., 2019). These results suggest that the dynamics of cell cycle status plays an important role in determining the regenerative and immunomodulatory effects exerted by UC-MSCs.

Molecular heterogeneity within the tissue extracted naïve UC-MSCs raise the intriguing question of whether UC-MSCs expanded *in vitro* prior to treatment retain the same stemness properties as their original isolates. In fact, single-cell RNA sequencing revealed three different subpopulations of UC-MSCs isolated from WJ, each expressing epithelial, pericyte, and stromal genetic markers, which defined their ligand-receptor crosstalk, as shown in Table 2. The epithelial subpopulation had immuno-modulatory, pro-survival, and differentiation potential, while the stromal subpopulation had properties more useful for regeneration and epithelial–mesenchymal transition (EMT). Molecular heterogeneity was revealed in single cell transcriptomics studies of WJ-MSCs *versus in vitro* clonally expanded UC-MSCs (Barrett et al., 2019; Zhang et al., 2021). Subpopulation changes affect gene expression patterns, possibly affecting the therapeutic effect of UC-MSCs. EMT genes, perhaps, were significantly expressed in tissue-derived UC-MSCs (Barrett et al., 2019). However, in the clonally expanded UC-MSCs, telomere maintenance associated DNA repair and senescence, required for irradiation-induced damage, was increased (Yang et al., 2016; Wu et al., 2017). This shows that even UC-MSCs from the same tissue have distinct ligand-receptor interactions that affect their biological activity in diverse disease microenvironments.

## Umbilical cord-mesenchymal stromal cell secretome and extracellular vesicles

Recent studies on cell-free therapy have suggested that the MSC secretome can also be used to treat different diseases (Eleuteri and Fierabracci, 2019; Soltani et al., 2021). Like other MSCs, UC-MSCs are reported to secrete molecules such as chemokines, cytokines, growth factors, and insoluble factors including EVs that have therapeutic effects in several diseases. The UC-MSC secretome consists of around 90 cytokines and numerous growth factors, which exhibit anti-inflammatory, pro-inflammatory, angiogenesis, neuroprotective, anti-apoptotic and anti-tumor properties (Bai et al., 2016). To investigate the therapeutic benefit of the UC-MSCs secretome, one study used injured brain extracts and pre-treated them with UC-MSCs; the secretome resulting from these pre-treated cells was then stereotaxically injected in rats with traumatic brain injury (TBI) with result showing a significant improvement in cognitive function when using the secretome from pre-treated UC-MSCs compared to the normal secretome (Liu et al., 2020a). In another study, the UC-MSC secretome was used in mice with diabetic wounds, and the results showed effective wound healing *via* an increase in angiogenesis as well as macrophage polarization from an inflammatory to an anti-inflammatory phenotype (Zhang et al., 2020).

International society for extracellular vesicles (ISEV) recognizes “EVs” as the general name for particles naturally expelled from the cell that are bordered by a lipid bilayer and cannot multiply (i.e. they do not include a functioning nucleus). EVs are described by 1) physical features such as size [100 nm or 200 nm (small) or >200 nm (big and/or medium)] or density (low, intermediate, high, with each range specified); 2) biochemical content (CD63^+^/CD81^+^ EVs, Annexin A5-stained EVs, *etc.*); and 3) conditions or cell of origin (podocyte EVs, hypoxic EVs, large oncosomes, apoptotic bodies) (Théry et al., 2018; Alcaraz et al., 2020). Haraszti *et al.* compared the yield of exosomes from three different types of MSCs such as AD-, BM-, and UC-MSCs and showed UC-MSCs to have the highest exosomal yield. Furthermore, modification of the EVs isolation method from differential ultra-centrifugation to tangential flow filtration and culture condition of UC-MSCs from 2D to 3D culture led to an increase in yield (Haraszti et al., 2018). In another study, UC-MSCs improved the cardioprotective effect in a MI model (Xu et al., 2020). Exosomes from UC-MSC have shown promising outcomes in the treatment of colitis (Ma et al., 2019), neurodegenerative diseases (Ding et al., 2018), liver failure (Jiang et al., 2019), diabetes (Zhang et al., 2020), as well as cancer (Ngadiono and Hardiany, 2019). ExoCarta, an online database is available to identify molecular markers of tissue/cell type derived exosomes, has catalogued various published and unpublished exosomal proteins, RNAs, and lipids. There are 100 genes encoding proteins in the ExoCarta, including the top five protein-coding genes (index>90) namely CD9, HSPA8, PDCD6IP, GAPDH, and Actin B.

Exosomes obtained from UC-MSCs have been reported to regulate inflammation and immune responses in acute liver failure (ALF) in mice models *via* reduction of NLRP3 inflammasome expression and downstream inflammatory cytokine secretion (Jiang et al., 2019). In addition, exosomes from TNF-α pre-treated UC-MSCs have efficiently prevented ALF by decreasing the NLRP3 expression and inflammatory factors (Zhang et al., 2020c). In another study, the delivery of UC-MSC EVs combined with estrogen exhibited a highly effective alternative treatment for endometrial injury (Ebrahim et al., 2018). Further, UC-MSCs exosomal microRNA-17–5P improved ovarian function by regulating SIRT7 expression following chemotherapy-induced damage in human ovarian cells (Ding et al., 2020). Hu *et al.* evaluated the effect of UC-MSC EVs in bone regeneration in osteoporotic mice and the study reported restoration of trabecular and cortical bone mass and attenuated bone loss in old mice. In another study, exosomes from UC-MSCs after osteogenic induction for 2 days were injected with hydrogel (hydroxylapatite embedded hyaluronic acid and alginate hydrogel) to provide long-term release of exosomes, which improved bone regeneration and angiogenesis (Yang et al., 2020). Jie *et al* evaluated the effect of UC-MSC exosomes on experimental colitis and showed significant improvement in the DSS-induced colitis in a mouse model (Ma et al., 2019). In the study by Shiue *et al*, constant intrathecal infusion of UC-MSC exosomes attenuated spinal nerve ligation-induced pain in rats. Moreover, the exosomes inhibited glial cell activation resulting in a decrease in proinflammatory cytokines (TNF-α, IL-1β) and an increase in the anti-inflammatory cytokines (IL-10) and neurotrophic factors (BDNF, GDNF) (Shiue et al., 2019). It has been reported that UC-MSC EVs protect HIE‐induced apoptosis by transferring microRNA (let‐7‐5p) to neuronal cells (Saeedi et al., 2019). Additionally, WJ-MSC EVs have been demonstrated to treat IR-induced kidney injury *via* regulation of ERK1/2 to suppress CX3CL1 to improve cell proliferation, inhibit apoptosis, and inflammation (Chen et al., 2017; Wu et al., 2018). Zhang *et al.* showed anti-oxidative regulation of UC-MSC EVs *via* downregulation of NOX2, and upregulation of NRF2/ARE in ischemia-reperfusion (IR)-induced kidney injured rats (Zhang et al., 2014; Zhang et al., 2016).

## Therapeutic potential of umbilical cord-mesenchymal stromal cells

**FIGURE 2:**
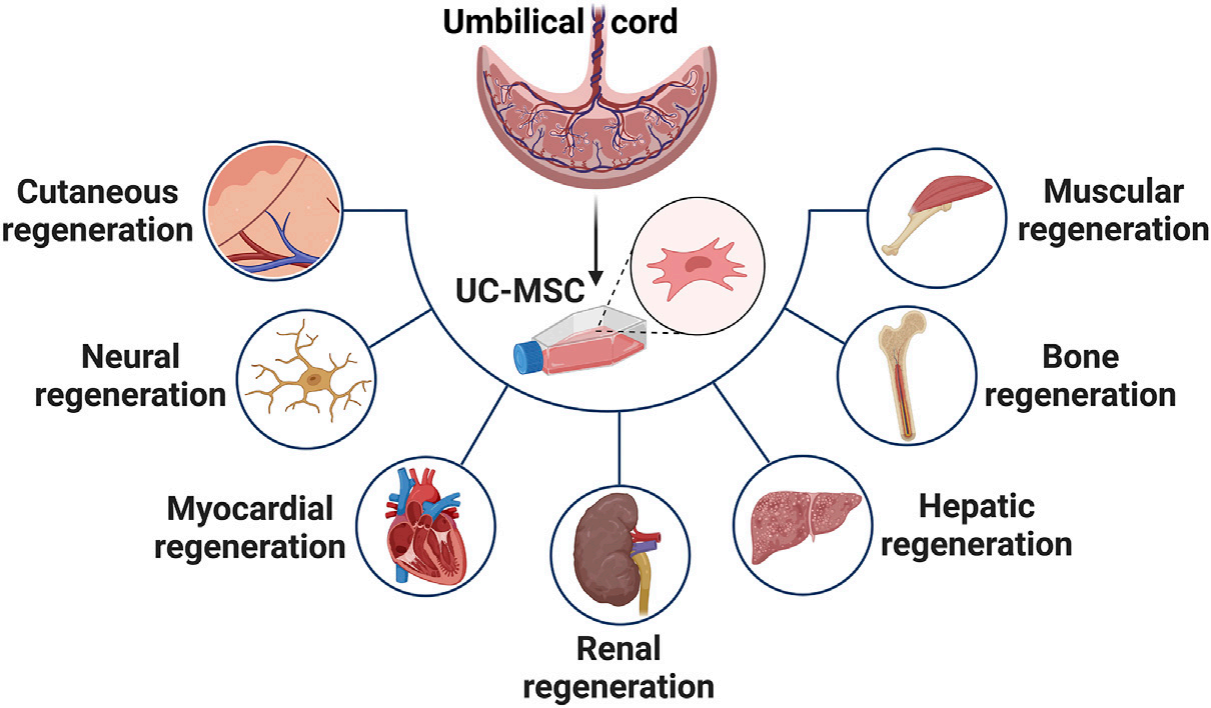
Highlights the applications of UC-MSCs for treatment of variety of diseases such as cutaneous, neural, myocardial, renal, hepatic, bone and muscular dysfunction.

MSCs have been shown to perform a regenerative function in damaged or injured tissues such as skin, bone, cartilage, liver, and cornea due to their abilities to secrete factors that directly induce tissue intrinsic progenitors, modulate local immune cells, inhibit apoptosis, prevent scar formation, and stimulate angiogenesis as illustrated in Figure 2 (Han et al., 2019) (Law and Chaudhuri, 2013).

### Neural regeneration

Ryu *et al.* demonstrated the paracrine potential of WJ-MSCs in promoting neural renewal, neural protection and preventing inflammation at the site of spinal cord injury (SCI), with evidence showing it to be more effective than BM- and AD-MSCs (Ryu et al., 2012). Mohamadi *et al.* have demonstrated anti‐inflammatory properties of WJ-MSCs following intrathecal administration in an SCI model (Mohamadi et al., 2019). WJ-MSCs have shown reduced expression of IL‐1b and increased expression of neural growth factor (NGF) in the treated spinal cord tissue; this results in renewal of motor function and integrity of spinal cord in a dose‐dependent manner, and over repeated administrations (Li et al., 2016; Chudickova et al., 2019). The therapeutic effects were further improved using conditioned media from WJ-MSCs (Chudickova et al., 2019). Recently, Wang *et al.* demonstrated enhanced functional retrieval, neural regeneration, and neurotrophic factors in the transected sciatic nerves of mice using WJ-MSCs in contrast to AD-MSCs (Wang et al., 2020). Also, Kim *et al.* have shown recovery of the behavioral role in hypoxic‐ischemic encephalopathy (HIE)-induced brain infarcted rats *via* anti-astroglia, anti-apoptotic, and anti-inflammatory factors of UC-MSCs (Kim et al., 2019). In another study, Lee *et al.* examined whether sRAGE secreting UC-MSCs *via* CRISPR/Cas9 technique protect neuronal cell death in a Parkinson’s Disease animal model (Lee et al., 2019). The study revealed that decreasing AGE-RAGE binding may be the potential therapeutic approach for curing Parkinson’s Disease by preventing neuronal cell death.

### Myocardial regeneration

The therapeutic effects of UC-MSCs in treatment of myocardial tissue is demonstrated in several studies. Liu *et al.* have shown improvement in left ventricular function, perfusion, and rejuvenation in a porcine model (Liu et al., 2016). UC-MSC treatment caused reduced expression of TNF-α and activation of Erk1/2 kinases in the heart tissue of rats cardiomyopathy, thereby preventing interstitial fibrosis and cardiac dysfunction (Zhang et al., 2018). Further, epigenetic modification of UC-MSCs have been shown to enhance cardiomyocyte differentiation through Wnt signaling in mice with cardiac injury (Bhuvanalakshmi et al., 2017). Rabbani *et al.* have proposed combinational therapy using WJ-MSCs with IGF-1 to regenerate myocardial tissue with enhanced angiogenesis, reduced fibrosis, and improvement in cardiac function in a rabbit model (Rabbani et al., 2017). This study revealed that overexpression of the anti-fibrotic factors in WJ-MSCs play an important role in protecting cardiac fibrosis owing to their greater viability, collagen deposition, and proliferation properties (Nimsanor et al., 2019) (Pu et al., 2017). Cho *et al.* investigated the cardio-protective benefit of UC-MSCs from rats expressing lymphoid enhancer-binding factor 1 (LEF1) linked using CRISPR/Cas9, and demonstrated increased cell survival and cardio-protective effects in the infarcted myocardium (Cho et al., 2020).

### Cutaneous regeneration

UC-MSCs have been widely explored as a potential alternative to other MSCs for regeneration of cutaneous tissues. Arno *et al.* have shown improved vascularization, re‐epithelialization, survival, proliferation, and migration of skin fibroblast cells in a skin-excised mouse model (Arno et al., 2014). Additionally, UC-MSCs have been reported to improve wound healing by supporting proliferation, angiogenesis, and regeneration of sebaceous glands in rats with radiation‐induced skin injury (Sun et al., 2019). The anti‐inflammatory potential was further enhanced by priming UC-MSCs with IFN-γ and poly (I:C) in atopic dermatitis (Park et al., 2019). Also, culturing of WJ-MSCs in a silk fibroin scaffold has shown improved re‐epithelialization and a reduction in the formation of fibrotic scars (Millán-Rivero et al., 2019). Recently, Martin‐Piedra *et al.* proposed that WJ-MSCs are better suited for preparation of bioengineered skin tissue over other types of MSCs such as AD-MSCs, dental pulp and BM-MSCs (Martin-Piedra et al., 2019).

### Hepatic regeneration

Mortezaee *et al.* reported retinoic acid as an effective inducer for stimulating the differentiation of WJ-MSCs into hepatocyte-like cells, though the exact mechanism remains incompletely understood. However, given that UC-MSCs do not readily differentiate *in vivo*, this approach will necessitate the *in vitro* generation and expansion of WJ-MSCs into hepatocyte-like cells prior to their administration with the additional need to also examine the expression of hepatocyte genes (Mortezaee et al., 2015). Tsai *et al.* demonstrated a reduction in serum glutamic oxaloacetic transaminase, TGF-β1, glutamic pyruvate transaminase, and collagen deposition, which cause an upregulation of mesenchymal‐epithelial transition factors and HG, leading to a decrease in liver fibrosis (Tsai et al., 2009). WJ-MSCs showed significant reduction of sepsis‐associated liver damage and endothelial dysfunction in a rat model (Cóndor et al., 2016). Interestingly, WJ-MSCs improved liver function and attenuated the d-Galactosamine-induced hepatotoxicity in acute liver injured mice (Ramanathan et al., 2017). Chetty *et al.* confirmed regeneration of liver in acetaminophen-induced injury *via* renewal of portal tracts and hepatic arteries after 3 days of WJ-MSCs administration in BALB/c mice (Chetty et al., 2019a). Another study showed paracrine activity of intrauterine transplanted WJ-MSCs in liver injured rabbit fetuses *via* expression of HNF-4, CYP2B6 messenger RNA, α‐fetoprotein, and albumin (Rezaeian et al., 2018). Hammam *et al.* observed that the combination of WJ-MSCs and praziquantel (PZQ) increased the therapeutic effects of PZQ on S. mansoni-induced liver fibrosis, as compared to utilizing each alone (Hammam et al., 2016).

### Renal regeneration

WJ-MSCs administration has revealed the role of endocrine effects in the treatment of IR-induced acute and chronic kidney injury through activation of Akt in tubular epithelial cells, resulting in inhibition of apoptosis and upregulation of HGF (Du et al., 2012). Hu *et al.* showed a reduction in renal fibrosis following administration of UC-MSCs in a decellularized kidney scaffold by inhibiting mesenchymal‐epithelial transition through TGF‐β/Smad pathway in rats (Hu et al., 2020). Several researchers have examined the use of UC-MSCs in kidney transplants from brain or cardiac deceased donors (Assfalg et al., 2016; Sun et al., 2018). These techniques are linked with a greater incidence of early graft malfunction and severe rejection due to prolonged ischemia (Summers et al., 2010). The combinatorial infusion of UC-MSCs before and during surgery in dead donor graft recipients was shown to be safe with no adverse clinical outcomes (Sun et al., 2017). Overall, UC-MSCs greater proliferative capacity, higher immunosuppressive effects, and hypo-immunogenic features make them suitable for large-scale, universal production.

### Cartilage regeneration

UC-MSCs have been shown to be an appealing source in combination with desired scaffolds for regeneration of cartilage tissue. Paduszyński *et al.* have shown regulation in the transcriptional profile of WJ‐MSCs following chondrogenic differentiation on a 3D Poly (lactic-co-glycolic acid) (PLGA) scaffold for repairing cartilage (Paduszyński et al., 2016). Similarly, several studies have demonstrated the therapeutic potential of UC-MSCs in combination with 3D HyStem and collagen-built hydrogel for cartilage repair due to elevation in the expression of cartilage‐specific matrix genes (Chen et al., 2013; Aleksander-Konert et al., 2016; Shie et al., 2017). Recently, Zhang *et al.* have described co-culture of UC-MSCs with articular cartilage cells in an extracellular matrix‐oriented scaffold for developing framework engineered hyaline cartilage *in vitro* (Zhang et al., 2019). In addition, UC-MSCs have revealed promising results in fabricating tissue‐engineered cartilage without using the scaffold. Saulnier *et al.* have reported that intra‐articular administration of WJ‐MSCs in the knee joints of 30 rabbits with osteoarthritis (OA) modulated MMP proteins in the synovium and prevented cartilage deprivation (Saulnier et al., 2015). Recently, Cheng *et al.* proposed a combinational therapy using WJ-MSCs along with shockwaves as an efficient treatment for early OA in rats (Cheng et al., 2019).

### Bone regeneration

Several studies have reported that UC-MSCs are a potential alternative to BM-MSCs for bone regeneration (Lim et al., 2018a; Ansari et al., 2018; Cabrera-Pérez et al., 2019). Todeschi *et al.* have reported enhanced angiogenesis after subcutaneous implantation of scaffolds loaded UC-MSCs in mice with bone defects (Todeschi et al., 2015). It has been hypothesized that the modulation in RUNX2/p57 expression *via* JARID1B histone demethylase inhibition plays a role in the osteogenic potential of WJ-MSCs (Bustos et al., 2017). Recently, Liu *et al.* have shown that overexpression of Wnt10b in UC-MSCs is beneficial for treatment of calvarial defect in rats due to an enhancement of osteogenic potential and VEGF-activated angiogenesis (Liu et al., 2020b). Choi *et al.* have recently shown that CRISPR-Cpf1 activation of the BMP4 gene promote osteogenic differentiation of UC-MSCs (Choi et al., 2020). Lastly, outcomes of phase I/II randomized control trials using UC-MSCs in patients after intra‐articular administration revealed the therapy to be more effective with repeated doses as compared to single-dose or hyaluronic acid treatment (Matas et al., 2019).

### Muscular regeneration

WJ-MSCs and their secreted XCL1 protein have been proposed as a novel approach for treating myopathies due to their anti-apoptotic potential (Kwon et al., 2016). Wang *et al.* have demonstrated a reduction in sarcopenia following activation of skeletal muscle cell proliferation, and an inhibition of apoptosis and inflammation in aged mice (Wang et al., 2018). Differentiation potential of WJ-MSCs into smooth muscle cells was shown to be accelerated by priming with TGF‐β1 and ascorbic acid (Mesure et al., 2017), and priming with SDF-1 improved the migration potential of UC-MSCs during regeneration of skeletal muscle *in vitro* (Kowalski et al., 2017). In addition, recovery of sensory and motor functions was enhanced *via* neuro‐muscular regeneration during neurotmesis injuries using biodegradable scaffolds loaded with UC-MSCs (Caseiro et al., 2017). Recently, Su *et al.* have demonstrated re-establishment of impaired skeletal muscle functions in mice *via* downregulation of neutrophil‐associated acute inflammation, attributed to the antifibrotic properties of UC-MSCs (Su et al., 2019).

## Biodistribution of umbilical cord-mesenchymal stromal cells

**FIGURE 3:**
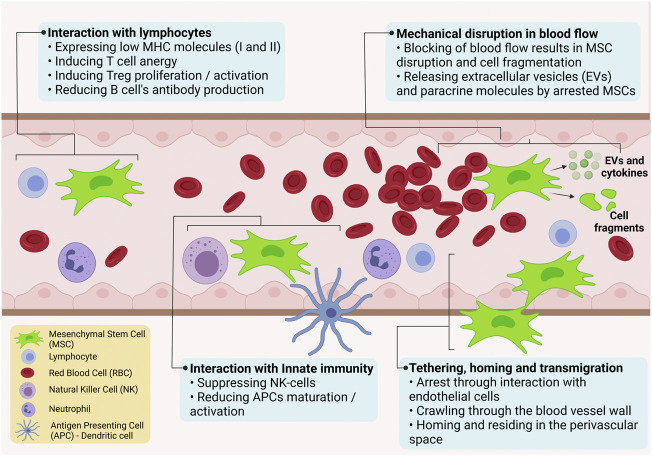
Schematic illustration of possible interactions of UC-MSCs following their systemic administration.

The most used delivery method for UC-MSCs in pre-clinical and clinical studies is systemic intravenous infusion of cells. In the last few years, labelling techniques have allowed scientists to detect the fate of MSCs after injection *in vivo* (Laurila et al., 2009; Reinders et al., 2013; Chetty et al., 2019b; Ullah et al., 2019). Lim *et al.* have reported that UC-MSCs transfected with lentivirus-eGFP were distributed in many organs, especially in the lungs after intravenous administration following acute myocardial infarction in porcine models (Lim et al., 2018b). Violatti *et al.* confirmed the previous results labelling UC-MSCs with poly (methyl methacrylate) nanoparticles loaded with the nuclear dye Hoechst-33258 for tracking in the healthy and early symptomatic SOD1G93A mice. Interestingly, the authors were able to track UC-MSCs at different time points during the disease progressions. In line with previous data, UC-MSCs, like other MSCs, are rapidly and efficiently captured by the lungs before reaching the reticuloendothelial organs (spleen, liver) for metabolism and clearance (Violatto et al., 2015). An outline of the potential interactions of UC-MSCs following their systemic administration is illustrated in Figure 3. T-cell anergy is caused by the lack of expression of the co-stimulatory molecules CD40, CD86, and CD80 on the surface of UC-MSCs. Allogeneic MSCs have been shown to inhibit the proliferation, activation, and IgG secretion of B cells from BXSB mice, which are used as an experimental model for human systemic lupus erythematosus. *In vitro* investigation also revealed that CD4^+^ T-cells in interaction with UC-MSCs are halted in the G0/G1 phase and cease to proliferate, whereas Treg cell proliferation is induced; plasma cell IgG production appears to be impacted as well (Nauta and Fibbe, 2007). Moreover, UC-MSCs express very little MHC-I and apparently no MHC-II (until after interferon therapy), making them resistant to NK cell toxicity in an allogenic/xenogeneic environment by down-regulating interferon expression and up-regulating the anti-inflammatory cytokines IL-4 and IL-10. Intravital microscopy of UC-MSCs in a cremaster muscle mouse model showed that UC-MSCs are likely to be disrupted by the shear force generated by blood flow. This can result in cell fragmentation and formation of extracellular vesicles capable of influencing paracrine secretion of immuno-modulatory factors, or trigger phagocytosis of the cell fragments by macrophages and endothelial cells. Following phagocytosis, immune modulatory factors are secreted, which then act to inhibit inflammation. UC-MSCs that survive the trip through the circulation may engage actively or passively with the endothelium wall, extravasate after engaging with the extracellular matrix (e.g., with MMP-2 and gelatinase), and eventually dwell in a pericyte-like position (Leibacher and Henschler, 2016).

## Clinical applications of umbilical cord-mesenchymal stromal cells

Numerous recent clinical trials have demonstrated the importance of UC-MSCs in the treatment of numerous diseases, as presented in Table 3 (Lv et al., 2013; Chang et al., 2014; Kim et al., 2015; Bartolucci et al., 2017; Riordan et al., 2018; Powell and Silvestri, 2019; He et al., 2020; Özmert and Arslan, 2020; Zhang et al., 2021; Kim et al., 2021; Lee et al., 2021; Shi et al., 2021; Monsel et al., 2022; Zang et al., 2022). We retrieved 119 studies using research requests citing “UC-MSCs, Umbilical Cord derived Mesenchymal stromal cells” on ClinicalTrials.gov, of which 36 are complete, 54 are recruiting, 13 are not recruiting, and 63 are in unclear inference. In brief, UC-MSCs’ immuno-modulatory, anti-inflammatory, and regenerative characteristics account for most of their therapeutic applications. The conclusions made as a result of these clinical trials are discussed below.

**TABLE 3:** Clinical Trials on UC-MSCs recruiting patients from ClinicalTrials.gov.

ClinicalTrials.gov identifier	Official title (status: Completed)	Sponsors and collaborators	Start date	Completion date
NCT02635464	The Safety and Efficacy Assessment of Human Umbilical Cord-derived Mesenchymal Stem Cells (hUC-MSCs) With Injectable Collagen Scaffold Transplantation for Chronic Ischemic Cardiomyopathy	Chinese Academy of Sciences and The Affiliated Nanjing Drum Tower Hospital of Nanjing University Medical School	October 2015	August 2019
NCT04288102	A Phase II, Multicenter, Randomized, Double-blind, Placebo-controlled Trial to Evaluate the Efficacy and Safety of Human Umbilical Cord-derived Mesenchymal Stem Cells in the Treatment of Severe COVID-19 Patients	Beijing 302 Hospital Huoshenshan Hospital Maternal and Child Health Hospital of Hubei Province and The General Hospital of Central Theater Command Vcanbio Cell & Gene Engineering Corp., Ltd., China	March 2020	July 2020
NCT04520022	Single Center, Single Group Assignment, Open Label Trial to Assess Safety and Effectiveness of Intravenous Allogeneic Umbilical Cord Blood-derived Mesenchymal Stem Cell in Patients with Recessive Dystrophic Epidermolysis Bullosa	Gangnam Severance Hospital and Daewoong Pharmaceutical Co. Ltd	October 2016	January 2020
NCT02034188	Feasibility Study of Human Umbilical Cord Tissue-Derived Mesenchymal Stem Cells in Patients with Multiple Sclerosis	Translational Biosciences	January 2014	March 2016
NCT04355728	Umbilical Cord-derived Mesenchymal Stem Cells for COVID-19 Patients with Acute Respiratory Distress Syndrome (ARDS)	Camillo Ricordi	April 2020	October 2020
NCT00823316	Phase 1/2 Study of Umbilical Cord Blood-Derived Mesenchymal Stem Cells Infusion for Promotion of Engraftment and Prevention of a Graft Rejection and Graft-versus-Host Disease After Unrelated Hematopoietic Stem Cell Transplantation	Medipost Co. Ltd	August 2008	February 2010
NCT02381366	Safety and Efficacy of PNEUMOSTEM^®^ in Premature Infants at High Risk for Bronchopulmonary Dysplasia (BPD) - a US Study	Medipost America Inc. and Medipost Co. Ltd	March 2015	May 2018
NCT01342250	Phase Ι/II Study of Human Umbilical Cord Mesenchymal Stem Cells Transplantation for Patients with Decompensated Liver Cirrhosis	Shenzhen Beike Bio-Technology Co., Ltd. and No.85 Hospital, Changning, Shanghai, China	October 2010	October 2011
NCT02291926	Phase I Study of Human Umbilical Cord Mesenchymal Stem Cell Implantation in the Treatment of Articular Cartilage Defect of Knee	Shenzhen Hornetcorn Bio-technology Company, Ltd. and The Fifth Affiliated Hospital of Guangzhou Medical University	December 2014	December 2016
NCT02235844	Allogeneic Transplantation of Human Umbilical Cord Mesenchymal Stem Cells (UC-MSC) for a Single Male Patient with Duchenne Muscular Dystrophy (DMD)	Allergy and Asthma Consultants, Wichita, Kansas and Aidan Foundation, Neil H. Riordan	September 2014	September 2017
NCT01739777	Phase 1 Randomized-Double Blind Clinical Trial of Intravenous Infusion of Umbilical Cord Mesenchymal Stem Cells Transplantation in Heart Failure on Patients with Cardiopathy in Dilated Stage, of Different Etiology	Universidad de los Andes, Chile	December 2012	June 2015
NCT02277145	Phase I Study of Radiation-induced Pulmonary Fibrosis Treated with Clinical Grade Umbilical Cord Mesenchymal Stem Cells	Jianwu Dai, Southwest Hospital, China	October 2014	December 2018
NCT02192749	Open, Prospective Trial of Treatment of Autism Spectrum Disorders (ASD) Using Intravenous Infusion of Umbilical Cord Tissue Mesenchymal Stem Cells (UC-MSC)	Translational Biosciences	July 2014	August 2017
NCT02481440	Repeated Subarachnoid Administrations of Human Umbilical Cord Mesenchymal Stem Cells in Treating Spinal Cord Injury	Limin Rong, Third Affiliated Hospital and Sun Yat-Sen University	March 2018	March 2020
NCT02302599	Efficacy and Safety of Umbilical-cord Mesenchymal Stem Cells in Chinese Adults with Type 2 Diabetes: A Single Center, Double-blind, Randomized, Placebo-controlled Trial	Chinese PLA General Hospital	January 2013	December 2020
NCT03724617	Clinical Study of Stem Cells in the Treatment of Thin Endometrium	Sir Run Shaw Hospital	October 2018	December 2020
NCT02668068	A Multicenter, Randomized, Single-blind, Parallel-group Study of Combined Large Volume WLL With Clinical Grade Umbilical Cord Mesenchymal Stem Cells (MSC) Transplantation for Treatment of Pneumoconiosis	Jianwu Dai Southwest Hospital, China and Nanjing Chest Hospital	January 2016	March 2019
NCT02669199	Between Umbilical Cord Mesenchymal Stem Cells Sources Sweat Gland Samples of Large Area Skin Wound Injury Patients Before and After the Transplant Center, Open, Random, Own More Controlled Clinical Trials	Chinese PLA General Hospital	January 2012	December 2015
NCT02685722	Umbilical Cord Mesenchymal Stem Cells Between Gel Treatment Difficult Skin Ulcer Healing Efficacy and Safety of Random, Open, Before-and-after Study	Chinese PLA General Hospital	January 2012	December 2015
NCT04219657	Comparison of Outcome of Mesenchymal Stem Cells and Skin Graft with Skin Graft in Management of Traumatic Heel Pad Injuries of Children	King Edward Medical University	October 2016	December 2019
NCT02054208	Safety and Exploratory Efficacy Study of NEUROSTEM^®^ *Versus* Placebo in Patients with Alzheimer’s Disease	Medipost Co. Ltd	March 2014	December 2019
NCT01343511	Phase Ι/Π Study of Stem Cell Therapy in Patients with Autism	Shenzhen Beike Bio-Technology Co., Ltd. and Shandong Jiaotong Hospital Association for the Handicapped of Jinan	March 2009	May 2011
NCT01297205	Safety and Efficacy Evaluation of PNEUMOSTEM^®^ Treatment in Premature Infants with Bronchopulmonary Dysplasia	Medipost Co. Ltd	December 2010	December 2011
NCT03337243	Effect of Implanting Allogenic Cytokines Derived from Human Amniotic Membrane (HAM) and Mesenchymal Stem Cells Derived from Human Umbilical Cord Wharton’s Jelly (HUMCWJ) on Pain and Functioning of Knee Osteoarthritis	Sport and Spine Rehab Clinical Research Foundation	November 2017	April 2019
NCT01873547	Different Efficacy Between Rehabilitation Therapy and Umbilical Cord Derived Mesenchymal Stem Cells Transplantation in Patients with Chronic Spinal Cord Injury in China	General Hospital of Chinese Armed Police Forces	June 2012	December 2015
NCT02378974	A Randomized, Double-blind, Placebo-controlled, Phase I/IIa Clinical Trial for Evaluation of the Safety and Potential Therapeutic Effects After Intravenous Transplantation of Human Umbilical Cord-derived Mesenchymal Stem Cells in Patients with Cerebral Infarction	CHABiotech CO., Ltd.	February 2015	April 2017
NCT04333368	Cell Therapy Using Umbilical Cord-derived Mesenchymal Stromal Cells in SARS-CoV-2-related ARDS (STROMA-CoV2)	Assistance Publique - Hôpitaux de Paris	April 2020	October 2021
NCT02755376	A Randomized, Single Center, Investigator Initiated Clinical Trial to Evaluate Enhancement of Healing Between Bone Tunnel and Graft in Anterior Cruciate Ligament (ACL) Injury Using Human Umbilical Cord Blood Derived Mesenchymal Stem Cell	Samsung Medical Center	January 2014	December 2018
NCT02644447	The Safety and Efficacy Assessment of Human Umbilical Cord-derived Mesenchymal Stem Cells (HUC-MSCs) With Injectable Collagen Scaffold Transplantation in Woman with Premature Ovarian Failure (POF)	Chinese Academy of Sciences and the Affiliated Nanjing Drum Tower Hospital of Nanjing University Medical School	October 2015	October 2018
NCT04224207	Management of Retinitis Pigmentosa by Wharton’s Jelly Derived Mesenchymal Stem Cells: Preliminary Clinical Results	Ankara Universitesi Teknokent	April 2019	January 2020
NCT01297218	Open-Label, Single-Center, Phase 1 Clinical Trial to Evaluate the Safety and the Efficacy of NEUROTSTEM®-AD in Patients with Dementia of the Alzheimer’s Type	Medipost Co. Ltd	February 2011	December 2011
NCT04522869	Umbilical Cord Derived Mesenchymal Stem Cell Transplantation for Children Suffering from Biliary Atresia	Vinmec Research Institute of Stem Cell and Gene Technology and Children’s Hospital, Vietnam	August 2019	October 2021
NCT05016011	Efficacy of Allogeneic UC-MSCs for Treating Large Defects Knee Injury	Cytopeutics Sdn. Bhd and Universiti Kebangsaan Malaysia Medical Centre	July 2020	June 2023
NCT03847844	UC-MSCs as Front-line Approach of Treatment for Patients With aGVHD (GVHD Cyto-MSC)	Cytopeutics Sdn. Bhd and Universiti Tunku Abdul Rahman		
Ministry of Science, Technology and Innovation, Malaysia	February 2019	December 2021		

### Umbilical cord-mesenchymal stromal cells are safe and well tolerated

A phase I/II, randomized, controlled study to assess the safety and efficacy of single/repeated intra-articular injection(s) of UC-MSCs in patients with knee osteoarthritis (NCT02580695) held by Universidad de Los Andes, Chile, showed no adverse events with a significant decrease in pain over 12 months and a reduction in cartilage degradation, inflammatory responses and bone sclerosis. Another phase I/II, an open-label dose escalation clinical trial on the safety and efficacy of intratracheal injection of UC-MSCs in premature infants with high risk for bronchopulmonary dysplasia (BPD) (NCT02381366) conducted by Medipost America Inc. Showed these cells to be well tolerated and safe in 12 extremely low birth weight infants (<28 weeks of gestation and <1,000 g at birth at 5–14 days) warranting for larger randomized-controlled blinded study.

UC-MSCs are clinically effective when given by different routes of administration. A randomized clinical trial of intravenously infused UC-MSCs in patients with cardiopathy (NCT01739777) held by Universidad de Los Andes, Chile demonstrated no adverse reactions to the cell infusion with no alloantibody development within the first 90-day post-treatment. Treated patients also showed significant improvement in left ventricular ejection fraction when assessed by transthoracic echocardiography and cardiac MRI. Another study examined the effects of peribulbar injection of UC-MSCs in patients with retinitis pigmentosa (NCT04315025; PT. Prodia StemCell Indonesia) and showed an improvement in the perception of light and vision, due to the UC-MSCs’ ability to regenerate new photoreceptors and retinal pigment epithelial cells.

### Umbilical cord-mesenchymal stromal cells can be disease modifying

A prospective, sequential, open-label phase III clinical study on sub-tenon administration of UC-MSCs for treating retinitis pigmentosa (RP) in patients (NCT04224207) was conducted by Ankara Universitesi Teknokent. Follow up of patients 12 months later demonstrated UC-MSCs is effective in the treatment of RP during the first year without any adverse effect by slowing or stopping the disease progression, regardless of the genetic mutation. In another completed trial, “Intravenously infused UC-MSCs treatment for Crohn’s disease” (NCT02445547) that was conducted by Fuzhou General Hospital showed a decrease in the Crohn’s disease activity, Harvey-Bradshaw index, corticosteroid dosage, and colonoscopy presented significant improvement in the mucosa in patients even at 12 months after receiving treatment.

### Umbilical cord-mesenchymal stromal cells can be safely used with bio-constructs

A study called “UC-MSCs gel treatment in difficult healing skin ulcers” (NCT02685722) was conducted by Chinese PLA General Hospital concluded the therapeutic effects of UC-MSCs were due to autophagy *via* clearance of advanced glycation end products at injured tissues. Another completed trial called “Clinical study of the treatment of infertility caused by recurrent intrauterine adhesions by collagen scaffold loaded with UC-MSCs” (NCT02313415) conducted by Nanjing University Medical School showed no treatment-related adverse effects, increased endometrial thickness due to endometrial proliferation, differentiation, neovascularization and decreased intrauterine adhesion score which raised the pregnancy above 38.4% by the end of a 30 months follow-up period.

## Banking of umbilical cord-mesenchymal stromal cells and ethical concerns

Large-scale growth of UC-MSCs *in vitro* is required since the number of newly separated cells from the cord tissue is so small (Nitkin and Bonfield, 2017). Thus, developing culturing methods that consistently produce clinical-grade UC-MSCs is critical. Apropos to this, bioreactors offer many benefits over regular two-dimensional culture process, including repeatability, simplicity of monitoring, quantity, and uniformity (Sensebé et al., 2013). Aseptic and automated measures, certified bioreactor additives, and tight monitoring are needed for large-scale production of clinical-grade UC-MSCs. Clinical applications benefit from the capacity to acquire UC-MSCs from donors, store them in the clinical-grade banks, and retrieve them as required. Cryopreservation media without serum has been utilized to prevent cells from being damaged by the freezing process (de Lima Prata et al., 2012). The rate of cooling affects MSC viability, and hence controlled rate freezers are often used for cryopreserving and maintaining UC-MSCs (Marquez-Curtis et al., 2015). Some of the companies that have established cutting-edge controlled rate freezers are listed in Table 4.

**TABLE 4:** Lists the companies involved in stem cell banking and treatment worldwide.

Company name	Description	Website
AMAG Pharmaceuticals, United States	Cord Blood Registry allows for the storage of new-born stem cells	https://www.amagpharma.com/
Cellular Dynamics International, United States	A Fujifilm subsidiary that operates an adult stem cell banking program and does research on bioprinting human tissues with stem cells	https://www.fujifilmcdi.com/
Cordlife, Singapore	It advertises itself as a cord blood stem cell storage insurance policy for new parents, and it claims to have completed 18 successful stem-cell infusions	https://www.cordlife.com/
Cordvida, Brazil	Offers cord blood stem cell cryopreservation and claims successful treatment of autism and cerebral palsy, with 8 stem-cell infusions utilized in transplants	https://www.cordvida.com.br/
Cryo Stemcell, India	Offers umbilical cord, platelet-rich plasma, and stem cell banking services to top eye hospitals, including cryopreservation of corneal stromal lenticules and limbal stem cell therapy	https://cryostemcell.in/index.html
Cryo-Cell International, United States	Offers a five-compartment cord blood collection kit for saving a new-born’s stem cells for utilization by more than one family member	https://www.cryo-cell.com/
Cryoviva India, India	Offers processing and storage of cord blood stem cells	https://cryoviva.in/
Global Cord Blood Corporation, China	Offers cord blood stem cell storage services throughout China and the Asia Pacific area	https://www.globalcordbloodcorp.com/
Insception Lifebank, Canada	The largest facility of its kind in Canada, the cord blood and tissue stem cell storage bank	https://www.insception.com/
LifeCell, India	Offers diagnostic prenatal screening and cryopreservation of cord blood stem cells	https://www.lifecell.in/
PerkinElmer, Massachusetts	Offers stem cell culturing and phenotypic screening in addition to cord blood stem cell banking	https://www.perkinelmer.com/
Reelabs, India	Offers stem cell banking from cord tissue, cord blood, amniotic sac and fluid, and placenta for autism, leukemia, liver cirrhosis, thalassemia, diabetes, osteoarthritis, and more	https://www.reelabs.com/
Smart Cells International, West Drayton, United Kingdom	Offers storage options for both infant and adult stem cells	https://www.smartcells.com/
Stemade Biotech, India	The first private dental stem cell bank in India, extracting stem cells from children’s primary teeth and wisdom teeth for therapeutic treatment	https://www.stemade.com/
StemCyte, California	A corporation that provides stem cell transplantation and therapy and a private cord blood bank	https://rwww.stemcyte.com/

When acquired safely and with a pre-agreement after delivery, the use of UC-MSCs do not raise ethical issues. These cells are now a crucial component of research and have shown to be effective as well as lifesaving in thousands of allogeneic settings. However, the storage of stromal cells by private, commercially managed banks for future autologous use for the child is a social and ethical issue. The European group on ethics (EGE) addressed this issue in its 16 March 2004: Opinion 19 on “Ethical considerations of umbilical cord blood banking” (https://ec.europa.eu/info/index_en). Only a few autologous applications are made so far for very uncommon illnesses that can be predicted based on the family history. In these situations, the clinic should preserve MSCs from umbilical cord as part of the solidarity system. In certain European countries (e.g., Italy), advertising for private and self-financed storage is prohibited, and lawsuits are being filed against this practice (e.g., in Belgium) (Virt, 2010). At present, it is essentially a matter of consumer protection, which entails safeguarding individuals from misleading expectations due to false advertising (CJT, 2013).

## Conclusion and future perspectives

The recent rise in interest in the unique characteristics of UC-MSCs generated from WJ is due to their advantages over BM-MSCs (the “classical” adult source of MSCs), and include their significant *in vitro* proliferation in addition to their paracrine and immunomodulatory capabilities. These characteristics, along with the ease with which UC-MSCs can be isolated from an endless supply of tissue, have sparked significant interest in their potential use in cell-based therapies supported by both pre-clinical and clinical investigations. However, sufficient comparative studies are needed soon to answer the long-standing issue of whether MSCs from various anatomical sites within the UC are associated with differences in their qualitative and quantitative therapeutic features. The global expression approaches such as multi-omics profiling, CGH microarrays, methylation mapping, CRISPR/Cas and lab-on-Chip technology are expected to significantly enhance the understanding of UC-MSC biology. These results should not only provide theoretical groundwork for future therapeutic applications of UC-MSCs, but also offer a major contribution to stromal cell biology in general.
